# Extracellular Vesicles Derived From Apoptotic Cells: An Essential Link Between Death and Regeneration

**DOI:** 10.3389/fcell.2020.573511

**Published:** 2020-10-02

**Authors:** Maojiao Li, Li Liao, Weidong Tian

**Affiliations:** ^1^Engineering Research Center of Oral Translational Medicine, Ministry of Education, West China Hospital of Stomatology, Sichuan University, Chengdu, China; ^2^National Engineering Laboratory for Oral Regenerative Medicine, West China Hospital of Stomatology, Sichuan University, Chengdu, China; ^3^State Key Laboratory of Oral Diseases, West China Hospital of Stomatology, Sichuan University, Chengdu, China; ^4^National Clinical Research Center for Oral Diseases, West China Hospital of Stomatology, Sichuan University, Chengdu, China; ^5^Department of Oral and Maxillofacial Surgery, West China Hospital of Stomatology, Sichuan University, Chengdu, China

**Keywords:** apoptosis, apoptotic cell derived extracellular vesicles, functional biomolecules, cell death, regeneration

## Abstract

Apoptosis is a universal and continuous event during tissue development, restoration, repair, and regeneration. Mounting evidence has demonstrated that apoptosis is essential for the activation of tissue regeneration. However, the underlying mechanism remains elusive. A striking development in recent years comes from research on extracellular vesicles (EVs) derived from apoptotic cells. During apoptosis, cells secrete vesicles of various sizes containing various components. Apoptotic cell-derived EVs (ApoEVs) have been found to transit to neighboring cells or cells in distant tissues through the circulation. These vesicles could act as containers to transmit the nucleic acid, protein, and lipid signals to target cells. ApoEVs have been shown to promote regeneration in the cardiovascular system, skin, bone, muscle, kidney, etc. Moreover, several specific signaling pathways mediating the anabolic effects of ApoEVs have been classified. In this review, we comprehensively discussed the latest findings on the function of ApoEVs in tissue regeneration and disease prevention. These findings may reveal unexpected clues regarding the regulatory network between cell death and tissue regeneration and suggest novel targets for regenerative medicine. The findings discussed here also raise the question whether and to what extent ApoEVs contribute to embryonic development. This question is all the more urgent because the exact functions of apoptotic events during numerous developmental processes are still largely unclear.

## Introduction

Apoptosis refers to the spontaneous and orderly cell death and is controlled by a cluster of genes to maintain homeostasis (Kroemer et al., 2005). It is estimated that over 50 billion cells undergo apoptosis in the human body every day to maintain homeostasis (Davidson and Steller, 1998; Pellettieri and Sanchez Alvarado, 2007; Bergmann and Steller, 2010; Fuchs and Steller, 2011; Hochreiter-Hufford et al., 2013; Arandjelovic and Ravichandran, 2015). Apoptosis is a common phenomenon in embryonic development, cell differentiation, tissue regeneration, and other physiological processes, as well as in tumor, immune deficiency, organ atrophy, and other pathological processes. During organogenesis, the coordination of apoptosis and mitosis can maintain a constant number of cells in tissues and organs to shape the normal embryos, especially in the CNS and in the immune system (Penaloza et al., 2006; Zakeri et al., 2015). Apoptosis also plays an important role in the sculpting and reconstruction of tissue morphology (Kroemer et al., 2005; Elmore, 2007). Certain differentiated cells are regularly eliminated and replaced by the progeny of adult stem cells to maintain anatomical structure and function, which is usually called the balance between death and regeneration. As a part of normal embryonic development and tissue homeostasis, apoptosis has been shown to play a pivotal role in balancing death and regeneration. During apoptosis, apoptotic cells secrete vesicles of various sizes named apoptotic cell-derived extracellular vesicles (ApoEVs). Emerging evidence has revealed that extracellular vesicles could carry beneficial substances and be essential for cell communication. ApoEVs could be engulfed by target cells and promote regeneration in skin, bone, muscle, etc., by transporting bioactive molecules (e.g., proteins, lipids, and nucleic acids). Although several reviews have summarized the role of ApoEVs in immune regulation and cancer development (Caruso and Poon, 2018; Gregory and Dransfield, 2018; Gregory and Paterson, 2018; Laurenzana et al., 2018; Muhsin-Sharafaldine and McLellan, 2018a,b; Pavlyukov et al., 2018; Grant et al., 2019; Battistelli and Falcieri, 2020), the emerging findings of ApoEVs in tissue regeneration and embryonic development have not been systematically reviewed. Here, we introduce the function of ApoEVs in tissue regeneration, embryonic development, and disease prevention and discuss the therapeutic benefits of ApoEVs in regenerative medicine.

## Apoptosis

### The Process of Apoptosis

In most cases, apoptotic cells show very similar patterns of morphological changes. The noticeable morphological changes associated with apoptosis are the shrinkage of the cell, condensation of the nucleus and chromatin, breakage of the nucleus and cytoplasmic organelles, and cell membrane detachment and blebbing (Hacker, 2000; Saraste and Pulkki, 2000; Kroemer et al., 2005). During this process, a series of membrane-wrapped structures named ApoEVs are formed through cell membrane budding and blebbing (Merchant et al., 2017), and then are engulfed and digested by target cells (Tower, 2015). The clearance of cellular “corpses” commences at the earliest stages of apoptosis. Timely and effective phagocytosis of apoptotic cells and their contents can hardly recruit inflammatory cells and is conducive to avoid inflammation (Arandjelovic and Ravichandran, 2015; Medina et al., 2020).

### The Mechanisms of Apoptosis

In 2012, the Nomenclature Committee on Cell Death (NCCD) initiated a new classification of cell death based on measurable biochemical characteristics, which divides the apoptotic pathway into intrinsic and extrinsic apoptosis.

#### The Intrinsic Apoptotic Pathway

The intrinsic apoptotic pathway is also known as the mitochondrial/cytochrome c pathway (Tower, 2015). Cytochrome c release from mitochondria is the key step. The decrease in mitochondrial transmembrane potential (Δψ) is the first step of mitochondrial-mediated apoptosis. Cellular stresses, including DNA damage, oncogene activation, hypoxia, and oxidative stress, leads to the opening of or damage to the mitochondrial permeability transition pore (MPTP), a decrease in Δψ, and an increase in mitochondrial permeability, which promote the release of cytochrome c and apoptosis-inducing factor (AIF) (Susin et al., 1999). Cytochrome c can recruit and activate Caspase-9. Thus, Caspase-9 activates caspase cascade reactions (Caspase-3, -6, and -7) to initiate irreversible caspase-dependent apoptosis (Slee et al., 1999; Joza et al., 2001; Elmore, 2007; Kuribayashi et al., 2014).

#### The Extrinsic Apoptotic Pathway

The extrinsic pathway is mediated by the death receptors of the tumor necrosis factor (TNF) receptor type 1 superfamily embedded on the cell membrane (Guicciardi and Gores, 2009; Tower, 2015). The binding of Fas ligand to Fas receptor recruits the adapter protein Fas-associated death domain protein (FADD), which recruits Caspase-8 zymogens to form the Caspase-8 activator named death-inducing signaling complex (DISC). Then the pro-Caspase-8/10 is activated through autocatalysis (Boatright and Salvesen, 2003), which initiates the apoptotic process (Elmore, 2007) and leads to the activation of the downstream effectors Caspases-3, -6, and -7 (Broker, 2005).

## Apoptosis Promotes Tissue Development and Regeneration

Apoptosis has been regarded as a critical control point in development. Apoptosis is essential for successful embryonic development and the maintenance of normal tissue homeostasis (von Mühlen and Tan, 1995; Rieux-Laucat et al., 2003; Gaipl et al., 2005; Macchi et al., 2015). In tissue development, apoptosis can sculpt structures, drive morphogenesis, regulate cell number, eliminate useless, potentially dangerous and senescent cells, and participate in the regulation of regeneration and differentiation (Penaloza et al., 2006; Fuchs and Steller, 2011; Zakeri et al., 2015). In addition, some findings have shown the link between cell death and tissue regeneration. As proposed by Kondo in 1988 (Kondo, 1988), apoptosis promotes cell proliferation during tissue regeneration (Kondo, 1988; Hwang et al., 2004; Li et al., 2004, 2010; Ryoo et al., 2004; Vlaskalin et al., 2004; Kondo et al., 2006; Tseng et al., 2007; Chera et al., 2009; Pellettieri et al., 2010), which is named “apoptosis-induced compensatory proliferation (AIP)” (Huh et al., 2004; Pérez-Garijo et al., 2004; Kondo et al., 2006; Fan and Bergmann, 2008). For example, in the mid-gastric amputation model, effector caspases trigger the release of the mitogen Wnt3 to stimulate proliferation and regeneration during apoptosis (Chera et al., 2009). Similarly, caspase activation promotes regeneration in the injured site in Xenopus tadpole tails after amputation (Tseng et al., 2007).

Apoptosis also subtly promotes cell differentiation. Caspase family activation in apoptosis is closely related to cell differentiation. For example, Caspases-3 and -9 promote monocyte differentiation to macrophages but not dendritic cells (Sordet et al., 2002). The absence of Caspase-8 leads to stagnation of macrophage differentiation. In stem cells (SCs), Caspase-3 can also mediate the differentiation of bone marrow stromal cells, neural SCs, embryonic SCs, and haematopoietic SCs (Feinstein-Rotkopf and Arama, 2009). The increased proliferation and regeneration can compensate for cell loss in the injured tissues (Brockes and Kumar, 2008).

Apoptosis can restore tissue regeneration by promoting the elimination of senescent cells (SnCs). SnCs accumulate in many vertebrate tissues with age and secret senescence-associated secretory phenotype (SASP) to inhibit regeneration (Downward et al., 2008; van Deursen, 2014). Jeon et al. (2017) found that selective elimination of SnCs attenuated the development of post-traumatic osteoarthritis (OA), reduced pain, and increased cartilage development. Intra-articular injection of a senolytic molecule that selectively killed SnCs promote repair and regeneration in aged mice. Thus, locally induced apoptosis of senescent cells is beneficial for tissue regeneration. In addition, apoptosis and cell senescence are complementary processes in the regression of embryonic tissues and share common regulatory signals. During the formation of free digits in the developing limbs, the Btg/Tob gene family is upregulated. Btg2 overexpression in mesodermal progenitors of the limbs increases oxidative stress and induces cell death and cell senescence, which inhibits limb outgrowth *in vivo* (Lorda-Diez et al., 2015).

## Apoptotic Cell-Derived Extracellular Vesicles

Apoptotic cell-derived extracellular vesicles (ApoEVs) are a group of subcellular membrane-bound extracellular vesicles generated during the decomposition of dying cells. ApoEVs can be generated by many types of cells, such as stem cells, immunocytes, precursor cells, osteoblasts, and endothelial cells (Jiang et al., 2017). At present, the classification of the ApoEVs is still controversial. Apoptotic bodies (ApoBDs) were the first identified ApoEVs (Ihara et al., 1998). However, with the development of detection technology, researchers have found smaller vesicles (Simpson and Mathivanan, 2012) produced by dying cells in addition to traditional apoptotic bodies. Although there is no well-defined criteria to distinguish ApoBDs from other ApoEVs, the vesicles can be classified by diameter: larger membrane-wrapped vesicles termed ApoBDs/ABs have diameters of 1000–5000 nm (Atkin-Smith et al., 2015), and the smaller vesicles termed apoptotic microvesicles (ApoMVs) or exosome-like ApoEVs (Park et al., 2018) have diameters of 50–1000 nm (Schiller et al., 2012; Ainola et al., 2018). Lacking standard classification makes it difficult to draw accurate conclusions on the functions of ApoEVs. In order to unify the classification, we re-summarize the subtypes of ApoEVs according to the size of the vesicles extracted by different isolation or characterization methods in Tables 1, 2.

**TABLE 1:** The function of ApoEVs in regeneration.

**Nomenclature used by the authors**	**ApoEVs types according to sizes**	**Original cell**	**Recipient cell**	**Diseases/experimental effect**	**Mechanism**	**Role of vesicles**	**References**
ApoBDs	Mix of ApoBDs and ApoMVs	Endothelial cells	Endothelial progenitor cells	Promote proliferation	Unclear	Unclear	Hristov et al., 2004
ApoBDs	Unclear	Cardiomyocytes	Cardiomyocyte precursors	Chronic post-infarction heart failure	Promote SC proliferation and differentiation	miRNA transfer?	Tyukavin et al., 2015
ApoBDs	ApoBDs	Macrophages	Epithelial cells	Promote proliferation	MiR-221 miR-222	miRNA transfer	Zhu et al., 2017
ApoBDs	Mix of ApoBDs and ApoMVs	Endothelial cells	Vascular cells	Atherosclerosis	Induced CXCL12-dependent vascular protection	miRNA transfer	Zernecke et al., 2009
ApoBDs	Mix of ApoBDs and ApoMVs	Epithelial stem cells	Epithelial stem cells	Promote proliferation and division	Activate Wnt8a	mRNA transfer	Brock et al., 2019
ApoBDs	ApoBDs	BMSCs	BMSCs	Osteopenia	Activate the Wnt/β-catenin pathway	miRNA transfer	Liu et al., 2018
ApoBDs	Mix of ApoBDs and ApoMVs	MSCs	Endothelial cells	Myocardial infarction	Regulate autophagy	Active lysosome function	Liu et al., 2020
CRK-MVs	ApoMVs	podocytes	Epithelial cells	Renal repair	Induce compensatory proliferation	Paracrine mediators?	Bussolati and Camussi, 2017
MOC- ApoBDs	ApoBDs	Osteoclasts	Pre-osteoblasts	Bone remodeling	Activate PI3K/AKT/mTOR/S6K signaling	RANK activator	Ma et al., 2019

*ApoBDs, apoptotic bodies; SCs, stem cells; BMSCs, bone mesenchymal stem cells; MSCs, mesenchymal stem cells; PI3K, phosphoinositide 3-kinase; AKT, AKT Ser–Thr kinase; S6K, ribosomal protein S6 kinase; RANK, nuclear factor kappa B.*

**TABLE 2:** The functions of ApoEVs in other diseases.

**Nomenclature used by the authors**	**ApoEVs types according to sizes**	**Original cell**	**Recipient cell**	**Diseases/experimental effect**	**Mechanism**	**Role of vesicles**	**References**
ApoEVs	ApoBDs	Tumor cells	DCs	Antitumour immunity	Induce CD8+ and CD4+ T cell response	Activate (MHC)-I and MHC-II pathways	Horrevorts et al., 2019
ApoBDs	Unclear	Leukemic B cells	DCs	B-CLL	Antitumour immunity	Antigen presentation	Hus et al., 2005
ApoEVs	ApoBDs	Melanoma cells	Injected into mice	Melanoma	Antitumour immunity	Antigen presentation	Muhsin-Sharafaldine et al., 2016
ApoEVs	ApoMVs	Macrophages	DCs	Tuberculosis infection diseases	Antimicrobial immunity	Antigen presentation	Winau et al., 2006
ApoBDs	Unclear	Prion-infected apoptotic neurons	DCs	Prion disease	Promote clearance of prion via Mfge8	Prion transfer	Kranich et al., 2010
ApoPMN-MY	ApoMVs	Neutrophil	CD25+Th cells	Maintain immunological tolerance	Suppress the proliferation of CD25+Th cell	IL-2 suppressor	Shen et al., 2017
ApoBDs	Unclear	β cells	DCs	Type 1 Diabetes	Immune tolerance	Antigen presentation	Marin-Gallen et al., 2010

*ApoEVs, apoptotic extracellular vesicles; DCs, dendritic cells; MHC, major histocompatibility complex; ApoBDs, apoptotic bodies; B-CLL, B-cell chronic lymphocytic leukemia; IL-2, Interleukin-2.*

How to identify and isolate ApoEVs remains a critical issue. During apoptosis, the most typical feature is the transfer of phosphatidylserine (PS) to the surface of the lipid layer, which is also characteristic of ApoEVs (Hengartner, 2001). The translocated PS binds to Annexin V, a 36 kDa calcium phospholipid-binding protein (Kurihara et al., 2008). *In vitro*, Annexin V is widely used to identify and image cell death and ApoEVs because of its high binding affinity with PS (Subiros-Funosas et al., 2017). Another membrane change is the oxidation of surface molecules, which helps the binding of thrombospondin (Tsb) and the complement protein C3b to the membrane (Wu et al., 2019) and, in turn, is recognized by the recipient cells. Therefore, Annexin V, Tsb, and C3b are well-accepted ApoEVs markers (Akers et al., 2013). Fluorescence-activated cell sorting (FACS) and differential centrifugation are the most commonly used methods to purify ApoEVs. FACS achieves a purity of up to 99%, while differential centrifugation achieves up to 95% purity (Phan et al., 2018). The flow cytometry-based method can effectively detect the contents and cell origins of ApoBDs (Jiang et al., 2017). However, FACS can only detect the large ApoBDs since most flow cytometers only detect micron-sized cells or vesicles and cannot distinguish ApoBDs and ApoMVs. Differential centrifugation can isolate ApoEVs of different sizes, but the purity is lower than that of FACS. This method may not be suitable for isolating ApoEVs from complex samples compared with FACS. The protocol only compares the purity of ApoEVs extracted by these two methods, but there has been no functional comparison of ApoEVs extracted by different methods. The fluorescent labeling technique with Trp-BODIPY cyclic peptide (Subiros-Funosas et al., 2017) and the *in situ* ligation technique (Hauser et al., 2017) may be emerging technologies for distinguishing ApoEVs from other vesicles. To progress the field, it is critical to identify suitable criteria to distinguish different subtypes of ApoEVs and develop better experimental systems to track ApoEV formation.

### The Formation of ApoEVs

The formation of ApoEVs can be divided into three key steps: (Step 1) membrane blebbing on the cell surface, which is now considered a prerequisite for the formation of ApoEVs (Lane et al., 2005); (Step 2) apoptotic membrane protrusions in the form of microtubule spikes, apoptopodia, and beaded apoptopodia, which secrete approximately 10–20 ApoEVs each time (Xu et al., 2019); and (Step 3) the formation of ApoEVs.

The production of ApoEVs is regulated in a dose- and time-dependent manner by different molecular factors, such as the Rho-associated protein kinase (ROCK1) (Coleman et al., 2001; Gregory and Dransfield, 2018; Aoki et al., 2020) and myosin-light chain kinase (MLCK) (Mills et al., 1998). Inhibitors of ROCK1, MLCK, and caspases can suppress this process. Functional microtubules help nuclear shrinkage, and MLCK contributes to the packaging of nuclear material into ApoEVs (Zirngibl et al., 2015). Actomyosin leads to an increase in cell contraction and hydrostatic pressure and the formation of blebs (Orlando et al., 2006). The plasma membrane channel pannexin 1 (PANX1) was recently described as a negative regulator of ApoBDs formation since trovafloxacin (a PANX1 inhibitor) promoted apoptotic cell disassembly (Poon et al., 2014a). However, the factors driving the formation of these individual ApoEVs is still unclear. The synergism of intracellular and extracellular factors is necessary for breaking apoptotic cells into individual vesicles, and some unknown elements separate membrane protrusions from the main cell body.

### ApoEVs Are Biological Vectors Carrying Functional Biomolecules

Extracellular vesicles (e.g., Exos and MVs) mediate intercellular communication by carrying signaling molecules (Buzas et al., 2014). ApoEVs envelop the remaining components of dead cells (Crescitelli et al., 2013), which include proteins (e.g., from the nucleus, mitochondria, and plasma membrane), lipids and nucleic acids (e.g., mRNA, long non-coding RNA, rRNA, miRNA, or fragments of these intact RNA molecules). ApoEVs have been found to act as containers to carry the remnants of their original cells to promote regeneration (Halicka et al., 2000). Horizontal transfer of DNA can occur between adjacent cells through ApoEVs. For example, the DNA contained in endothelial cell-derived ApoBDs can induce the proliferation and differentiation of human endothelial progenitor cells *in vitro* (Hristov et al., 2004). DNA packaged into lymphoma-derived ApoBDs can be engulfed by fibroblasts, resulting in gene recombination (Holmgren et al., 1999). By shuttling microRNA-221/222, macrophage-derived ApoBDs promote the proliferation of lung epithelial cells (Zhu et al., 2017). MicroRNAs enclosed in ApoBDs from cardiomyocytes enhance the proliferation and differentiation of resident SCs *in vitro* (Tyukavin et al., 2015). Mesenchymal stem cells (MSCs) can engulf ApoBDs and reuse ApoBD-derived ubiquitin ligase RNF146 and miR-328-3p to promote bone regeneration (Liu et al., 2018). However, in Kogianni et al. (2008) showed that dying osteocytes release ApoBDs containing receptor activator of nuclear factor kappa-B ligand (RANKL) to recruit replacement osteoclasts, which can initiate osteoclastogenesis and localized bone destruction. Administration of ApoBDs carrying miR-126 inhibits atherosclerosis and induces CXCL12-dependent vascular protection (Zernecke et al., 2009). Besides, endothelial cell-derived miR-126 is transferred in ApoMVs to promote vascular regeneration and prevent atherogenesis (Nazari-Jahantigh et al., 2012). Interestingly, DNA and RNA cannot be simultaneously packaged into ApoBDs derived from HL-60 cells. Over 90% of ApoBDs containing RNA did not contain DNA, and vice versa, ApoBDs containing DNA did not contain RNA, which suggests that some biomolecules may selectively enter ApoEVs (Halicka et al., 2000). Human primary monocyte-derived macrophages can engulf ApoBDs containing autoantigens, suggesting that ApoEVs can play a role in antigen presentation in immunoregulation (Tran et al., 2002). Compared with the secretion of cytokines and growth factors, this mode of intercellular communication circumvents the “signal dilution effect” of soluble products released from apoptotic cells. Bioactive molecules entering the vesicles can act effectively on adjacent cells or distant tissues through circulation.

### ApoEVs Can Be Recognized and Engulfed by Target Cells

Several signaling molecules expressed on ApoEVs can be recognized by target cells. Segundo et al. (1999) proposed that apoptotic blebs recruit macrophages to death sites. This study showed that many CD molecules (including CD11a, CD21, CD22, and CD54) were partly released from germinal center B cells in the form of ApoEVs, which stimulated the chemotaxis of human monocytes.

The inversion of PS and the change in oxidation of surface molecules also contribute to the recognition of ApoEVs. Besides, CX3CL1 and ICAM-3 are potential recognition proteins on ApoEVs. CX3CL1, an intercellular adhesion molecule known as a “find-me” signal, is expressed on ApoEVs and can be recognized by the CXCL1 receptor (CXCL1R) expressed on mononuclear phagocytes. The interaction between CX3CL1 and CX3CR1 induces the migration of macrophages to apoptotic Mutu-BL cells (Truman et al., 2008). ICAM-3, an intercellular adhesion molecule expressed on the ApoEVs derived from leukocytes, can attract macrophages to induce leukocyte death (Torr et al., 2012). In summary, signal molecules embedded on the surface of ApoEVs may act as signals for engulfing, transferring, and mediating intercellular communication.

## The Function of Apoptotic Extracellular Vesicles in Tissue Regeneration

The transport of functional molecules through vesicles can be a new way to promote cell proliferation, tissue regeneration, and repair. Since ApoEVs can transmit bioactive molecules into neighboring cells or the cells in distant tissues through circulation, they play an active role in maintaining homeostasis after being phagocytized. ApoEVs produced by different cells have different effects on physiological and pathological processes. Here, we discuss the role of ApoEVs in tissue regeneration and disease treatment.

### ApoEVs Trigger the Clearance of Damaged or Senescent Cells

Apoptotic cells are primarily engulfed and cleared by phagocytes, which is of considerable significance to the dynamic equilibrium and immune response of healthy tissues. The prompt removal of dead cells by phagocytes can prevent cytotoxic substances or antigens from leaking into surrounding tissues (Poon et al., 2014b), effectively leading to the maintenance of tissue homeostasis. This process can be divided into three stages: recruitment, identification, and phagocytosis (Gardai et al., 2006; D’mello et al., 2009; Ravichandran, 2011).

**FIGURE 1:**
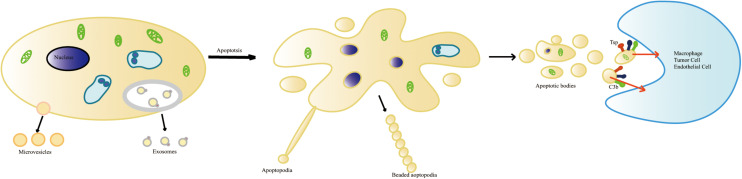
The formation of apoptotic cell-derived extracellular vesicles during apoptosis. Healthy cells generate two types of vesicles: exosomes and microvesicles. Apoptotic progress includes several stages: shrinkage of the cell, the condensation of the nucleus and chromatin pyknosis or karyorrhexis, the breaking of the nucleus, and cytoplasmic organelles and cell membrane detaching and blebbing. The disintegration of the cellular content into distinct membrane-enclosed vesicles termed apoptotic cell-derived extracellular vesicles (ApoEVs). ApoEVs can be engulfed by different cells mediated by specific recognition between recognition receptors and ligands. During apoptosis, the surface of the ApoEVs reveals specific changes. These changes include phosphatidylserine ectropion to bind with Annexin V and oxidation of surface molecules to bind with Thrombospondin (Tsp) or the complement protein C3b.

Recipient cells recognize “eat-me” signals such as PS on ApoEVs and initiate the engulfment process (Peter et al., 2008; Battistelli and Falcieri, 2020; Figure 1). For example, dendritic cells (DCs) engulf glycan-modified melanoma-derived ApoEVs carrying dendritic cell-specific intercellular adhesion molecule-3-grabbing non-integrin (DC-SIGN) ligands (Horrevorts et al., 2019). Apoptosis induces the production of neuronal apolipoprotein-E (apoE), which helps to clear ApoBDs via apoE–receptor interactions (Elliott et al., 2007). Microglial cells phagocytose and degrade apoptotic material from ApoBDs via microglial lysosomal and proteasomal pathways mediated by the CD36 scavenger receptor (Stolzing and Grune, 2004). Suppression of the formation of ApoBDs impairs the clearance of apoptotic or damaged cells by monocytes and macrophages (Orlando et al., 2006; Witasp et al., 2007), suggesting that ApoEVs can effectively promote the clearance of apoptotic cells.

### ApoEVs and Cell Survival, Proliferation, and Differentiation

Apoptotic cell-derived EVs are closely related to cell survival, proliferation, and differentiation. For example, ApoBD-rich medium (ApoBD-RM) from endothelial cells (ECs) promotes the proliferation of human endothelial progenitor cells (EPCs) *in vitro*, whereas ApoBD-depleted medium does not. ApoBDs engulfed by EPCs enhance EPC proliferation and differentiation (Hristov et al., 2004), which may contribute to the repair of damaged endothelial cells.

In addition, ApoBDs from cardiomyocytes enhance the proliferation and differentiation of resident stem cells (SCs) by transporting specific miRNAs (Tyukavin et al., 2015). Macrophage-derived ApoBDs promote the proliferation of recipient epithelial cells through ApoBD shuttling of miR-221 and miR-222 (Zhu et al., 2017). Endothelial cell-derived ApoBDs facilitate the survival of recipient vascular cells (human umbilical vein endothelial cells, mouse aortic endothelial cells, and smooth muscle cells) to survive. Selective enrichment of miR-126 in apoptotic endothelial cell-derived microvesicles stimulates the production of the chemokine CXCL12 in target cells, suppressing progenitor cell apoptosis (Zernecke et al., 2009).

The Wnt/β-catenin signaling pathway is a group of evolutionarily conserved signals that are essential in embryonic development and tissue regeneration. In a zebrafish model, ApoBDs containing Wnt-8a from dying SCs can promote the proliferation and regeneration of living epithelial cells and the communication with adjacent SCs. After phagocytizing ApoBDs, the Wnt signaling is activated to stimulate the division of basal SCs to maintain the total number of basal SCs in the tissue (Brock et al., 2019). ApoBDs activate the Wnt/β-catenin pathway to promote bone marrow mesenchymal stem cell (BMSC) proliferation and osteogenic, and lipogenic differentiation in Fas-deficient MRL/lpr and Caspase 3^–/–^ mice (Liu et al., 2018). Thus, activating the Wnt pathway via ApoEVs is essential for promoting cell proliferation and differentiation.

### ApoEVs Promote Tissue Development and Regeneration

#### ApoEVs Promote the Regeneration of the Cardiovascular System

Mesenchymal stem cells-derived ApoBDs engulfed by recipient endothelial cells (ECs) promote angiogenesis and cardiac functional recovery in a rat myocardial infarction (MI) model by regulating autophagy (Liu et al., 2020). Cardiomyocyte-derived ApoBDs stimulate the proliferation and differentiation of cardiomyocyte precursors. In a Wistar rat heart failure model, cardiomyocyte-derived ApoBDs improve the heart’s systolic function during the early apoptotic period (Tyukavin et al., 2015). In addition, human endothelial cell-derived ApoMVs carrying miR-126 upregulate CXCL12 in vascular cells, which recruits the incorporation of Sca-1+ progenitor cells to promote vascular protection and inhibit atherosclerosis (Zernecke et al., 2009). These findings underscore the functions of ApoEVs in cardiovascular regeneration.

#### ApoEVs Promote the Regeneration of the Kidney

CRK-containing microvesicles (CRK-MVs), a kind of ApoMV found in damaged glomeruli (Gupta et al., 2017), have been found to promote kidney regeneration. Apoptotic podocyte-derived ApoMVs might induce compensatory proliferation of parietal epithelial cells and injured tubular epithelial cells. Notably, ApoMVs induce compensatory proliferation in most of the analyzed target epithelial cells (Gupta et al., 2017). Apoptotic MSC-derived EVs act as paracrine signals to promote the repair of nephrons after injury (Bussolati and Camussi, 2017).

#### ApoEVs Promote Bone Regeneration

**FIGURE 2:**
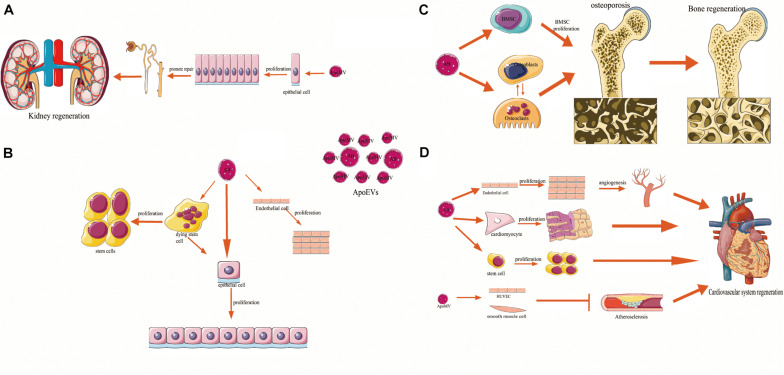
The relation of apoptotic cell-derived extracellular vesicles (ApoEVs) to cell survival, proliferation, differentiation, and tissue regeneration. ApoEVs can be divided into two groups: the larger apoptotic bodies (ApoBDs, ABs) and the smaller apoptotic microvesicles (ApoMVs). **(A)** ApoMVs induce compensatory proliferation of parietal epithelial cells and injured tubular epithelial cells, thus helping nephron repair. **(B)** *In vitro* experiments, ApoBDs engulfed by human endothelial cells, and epithelial cells can promote the proliferation of their recipient cells, respectively. Besides, ApoBDs from dying stem cells can promote the proliferation and regeneration of living epithelial cells and the communication with adjacent stem cells. **(C)** Bone marrow mesenchymal stem cells and osteoblasts have been found to engulf ApoBDs. Exogenous ApoBDs reversed the MSCs damage and improved the osteopenia, which can promote bone regeneration. Besides, ApoBDs can mediate intercellular communication signal mechanisms in osteoclast–osteoblast, suggesting its function in bone regeneration. **(D)** Endothelial cells (ECs) can engulf ApoBDs and promote angiogenesis and cardiac functional recovery. ApoBDs stimulated proliferation and differentiation of cardiomyocyte precursors and resident stem cells (SCs). ApoMVs transfer signals to vascular cells, induce vascular protection and inhibit atherosclerosis, which underlines the functions of ApoEVs in regeneration of the cardiovascular system.

Osteoporosis is a kind of systemic osteopathy caused by the imbalances in bone formation and bone resorption. Cerri (2005) showed that osteoblasts engulf bone cell-derived ApoBDs during the rat maxilla alveolar bone formation. Recently, (Liu et al., 2018) showed that systemic injection of exogenous BMSC-derived ApoBDs reversed MSC damage and improved the osteopenia in ovariectomized (OVX) mice. MSCs can engulf ApoBDs via integrin αvβ3 and inhibit Axin. The ApoBD-derived ubiquitin ligases RNF146 and miR-328-3p, thus activate the Wnt/β-catenin pathway. This finding confirms the role of ApoBDs in maintaining bone homeostasis and suggests the potential of ApoBDs in treating osteoporosis. ApoBDs also mediate intercellular communication between osteoclasts and osteoblasts. In bone remodeling, mature osteoclast-derived ApoBDs (mOC-ApoBDs) are taken up by pre-osteoblastic cells and prompt osteogenic differentiation by activating phosphoinositide 3-kinase (PI3K)/AKT Ser–Thr kinase (AKT) signaling (Ma et al., 2019; Table 1 and Figure 2). Moreover, previous studies suggested that osteocyte-derived ApoBDs could recruit the osteoclasts and initiate local bone resorption (Yuan et al., 2018), suggesting that ApoBDs derived from different sources play distinct roles in bone remodeling.

#### ApoEVs During Embryonic Development

Apoptosis can be detected at many stages of mammalian early embryonic development. In the process of embryo formation and development, apoptosis can remove abnormal and redundant cells. Apoptosis in mammalian blastocysts is very important for the further development of normal embryos (Hardy, 1999; Fabian et al., 2005; Jezek and Kozina, 2009). During embryonic development, apoptosis can help systems matching, sculpt the body, remove the outlived sculpture, and protect the organism, especially in neurulation, eye or ear development/invagination, limb modeling, development of the immune system (Cohen, 1999; Buss et al., 2006; Lorda-Diez et al., 2015; Hosseini and Taber, 2018). Jurisicova et al. (1996) found that a large number of ApoBDs in fragmented human embryos during embryonic development has increased since the blastocyst stage (Fabian et al., 2005). In limb modeling, dying cells generate ApoBDs which are engulfed by macrophages (Penaloza et al., 2006).

Since ApoEVs could affect the proliferation and differentiation of stem cells in adult tissue, it is highly probable that a large number of ApoEVs also play irreplaceable roles in embryonic development. Until now, there has been little evidence on whether ApoEVs participate in the formation and development of embryos directly. Further investigations are necessary to uncover the possible roles of ApoEVs in embryonic development.

## The Potential of ApoEVs in Disease Treatment

Based on the importance of ApoEVs in tissue regeneration, ApoEVs have been applied in the treatment of several diseases including degenerative diseases, tumors, and inflammatory diseases.

### ApoEVs in Degenerative Disease Treatment

In the cardiovascular system, ApoBDs from MSCs can promote angiogenesis and the heart’s systolic function recovery to prevent myocardial infarction via the regulation of autophagy (Tyukavin et al., 2015; Liu et al., 2020). Cardiomyocyte-derived ApoBDs revive cardiomyocyte precursors, leading to the alleviation of heart failure in the early stage. Endothelial cell-derived ApoMVs can protect the vasculature and inhibit atherosclerosis by transferring miRNAs (Zernecke et al., 2009). In the urinary system, the ApoMVs can restore injured tubular epithelial cells and facilitate nephron repair via compensatory proliferation (Gupta et al., 2017). In bone diseases, BMSC-derived ApoBDs can maintain bone homeostasis and treat osteoporosis. Thus, ApoEVs may be a new tool for degenerative disease treatment. However, the above findings are limited to the experimental stage. Extensive study of the mechanism and effect of ApoEVs in the degenerative disease treatment are necessary before the clinical application.

### ApoEVs in Tumor Treatment

Apoptotic cell-derived EVs from tumor cells have been shown to initiate antitumour immunity (Horrevorts et al., 2019). ApoEVs transfer pathogen carcinogens to antigen-presenting cells and protect the host from the tumor (Horrevorts et al., 2019). ApoEVs derived from tumor cells contain a high level of mannose glycans. These EVs can be engulfed by dendritic cells (DCs) more easily via dendritic cell-specific intercellular adhesion molecule-3-grabbing non-integrin (DC-SIGN). These DCs then act through both major histocompatibility complex (MHC)-I and MHC-II pathways to induce CD8^+^ and CD4^+^ T cell responses. It has been shown that allogeneic DCs engulfing ApoBDs from leukemic B cells stimulate antitumour immunity in B-cell chronic lymphocytic leukemia (B-CLL), suggesting that ApoBDs can serve as vaccines in tumor immunotherapy (Chang et al., 2000; Hus et al., 2005). ApoEVs derived from apoptotic melanoma cells (B16-OVA cells) initiate antitumour immunity and protect mice against subsequent tumor progression. The tumor antigen PMEL was found in ApoEVs and T cells, confirming that ApoEVs facilitate the transport of tumor antigens to antigen-presenting cells to promote antitumour immunity (Muhsin-Sharafaldine et al., 2016). Interestingly, although the PMEL in ApoEVs was lower than that in other vesicles, the antitumour protective effect of ApoEVs was more significant, suggesting that ApoEVs work through a different mechanism. Taken together, these findings indicate that ApoEVs can act as mediators of intercellular communication in the tumor. Specific ApoEVs may be useful biomarkers in monitoring disease progression. But novel test methods to detect ApoEVs and their cargos efficiently and accurately are necessary.

### ApoEVs in Immune and Inflammatory Disease Treatment

Apoptotic cell-derived EVs from apoptotic macrophages infected with *M. tuberculosis* can be engulfed by dendritic cells derived from peripheral blood mononuclear cells and splenic dendritic cells. Antigens on the ApoEVs are then presented to naïve CD4^+^ or CD8^+^ T cells to trigger antimicrobial immunity and eliminate *M. tuberculosis*, suggesting the potential use of ApoEVs as a vaccine (Winau et al., 2006). Whether ApoEVs can play a role in regulating the antimicrobial immunity against other pathogens remains to be explored. ApoBDs derived from prion-infected apoptotic neurons can be engulfed by microglia, which suppresses prion disease by promoting prion clearance via astrocyte-borne Mfge8 (milk fat globule epidermal growth factor 8) (Kranich et al., 2010; Tait and Green, 2010). Besides, apoptotic human polymorphonuclear neutrophil microvesicles (apoPMN-MVs, a kind of ApoMVs) selectively suppress the proliferation of CD25^+^ T_h_ cells in a dose-dependent manner by downregulating IL-2 and IL-2R expression. This downregulation inhibits the activation of resting T cells, thereby maintaining immunological tolerance (Shen et al., 2017). Immature DCs derived from the bone marrow of non-obese diabetic mice engulf antigen-specific apoptotic bodies from β cells. These DCs reduce the secretion of proinflammatory cytokines, and prevent experimental type 1 diabetes, suggesting that antigen-specific ApoBDs engulfed by DCs play an essential role in immunosuppression (Marin-Gallen et al., 2010). However, whether apoptotic β cell-derived ApoBDs prevent diabetes by promoting islet cell regeneration needs further exploration (Table 2). Although ApoEVs can present antigen and facilitate immune defense response in some cases, ApoEVs can also act as an autoantigen to induce autoimmune diseases, such as systemic lupus erythematosus (Cocca et al., 2002), suggesting that ApoEVs may play a completely different role in different environments.

## Concluding Remarks and Perspectives

Regeneration and embryonic development are partly based on common regulatory gene networks which, in both cases, may drive similar or even identical apoptosis and/or senescence processes. Apoptosis is a critical process in embryogenesis and postnatal cell homeostasis by balancing proliferation and death. Apoptosis accompanies the generation of membranous vesicles termed apoptotic extracellular vesicles (ApoEVs). ApoEVs can transit to the target cells and exchange signaling molecules, including DNA, RNA, and proteins, to regulate cell proliferation and differentiation and tissue regeneration after phagocytosis. Overall, there is compelling evidence to support the importance of ApoEVs in regulating tissue development and regeneration, such as in the cardiovascular system, urinary system, and bone. ApoEVs are potential components in the treatment of tumors, inflammatory diseases, and degenerative diseases. Therefore, the neglected ApoEVs could be considered as a key mechanism of intracellular communication. The ability of ApoEVs to proliferate and differentiate demonstrates a good balance between the beneficial effects of apoptosis and regeneration. Although there have been very few studies about the ApoEVs and their particular developmental processes, it is necessary to further explore the relationship between them. Compared with traditional drugs, ApoEVs have several advantages: (1) ApoEVs can be easily recognized by target cells through specific markers (PS, Tsb, and C3b); (2) the bioactive factors enveloped in ApoEVs provide essential signals to simultaneously promote the various functions of cells; (3) ApoEVs can affect adjacent tissues or distant tissue through the circulation. Emerging evidence has shown that ApoEVs are useful tools in tissue regeneration and disease treatment. Nevertheless, there remain several hurdles and challenges to be overcome before clinical applications of ApoEVs in disease treatment and tissue regeneration. Several critical questions need to be answered: How can ApoEVs play a decisive role in particular types of diseases? Do ApoEVs work by transferring specific contents directly or indirectly by recruiting cells or factors? How can the release, size, and specific cargo of ApoEVs be controlled? Is the formation of ApoEVs selective or cell-dependent? How can the transfer of bioactive molecules in ApoEVs be regulated? Is there a new way to distinguish different subtypes of ApoEVs? By addressing these questions, we will take a step closer to understanding ApoEVs in physiological and pathological conditions.

## Author Contributions

ML was responsible for collecting and collating documents and writing this review. LL and WT were responsible for writing and proofing the manuscript. All authors read and approved the final manuscript.

## Conflict of Interest

The authors declare that the research was conducted in the absence of any commercial or financial relationships that could be construed as a potential conflict of interest.
